# Genotype-specific and cross-reactive neutralizing antibodies induced by dengue virus infection: detection of antibodies with different levels of neutralizing activities against homologous and heterologous genotypes of dengue virus type 2 in common marmosets (*Callithrix jacchus*)

**DOI:** 10.1186/s12985-018-0967-x

**Published:** 2018-03-27

**Authors:** Nor Azila Muhammad Azami, Meng Ling Moi, Yasushi Ami, Yuriko Suzaki, Chang-Kweng Lim, Satoshi Taniguchi, Masayuki Saijo, Tomohiko Takasaki, Ichiro Kurane

**Affiliations:** 1 Department of Virology 1, National Institute of Infectious Diseases, Tokyo, Japan; 2 Graduate School of Comprehensive Human Sciences, University of Tsukuba, Ibaraki, Japan; 3 Institute of Tropical Medicine, Nagasaki University, Nagasaki, Japan; 4 Division of Experimental Animal Research, National Institute of Infectious Diseases, Tokyo, Japan; 5 Kanagawa Prefectural Institute of Public Health, Kanagawa, Japan; 6 National Institute of Infectious Diseases, 1-23-1 Toyama, Shinjuku, Tokyo, 162-8640 Japan

**Keywords:** Dengue, Antibody response, Genotype, Animal model

## Abstract

**Background:**

A vaccine against all four dengue virus (DENV) serotypes includes the formulation of one genotype of each serotype. Although genetic similarities among genotypes within a serotype are higher as compared to those among serotypes, differences in the immunogenicity of the included genotypes would be a critical issue in maximizing successful dengue vaccine development. Thus, we determined the neutralizing antibody responses against three genotypes of dengue virus serotype 2 (DENV-2), namely Cosmopolitan, Asian I, and Asian/American, after primary and secondary inoculation with DENV-2 in a dengue animal model, the common marmoset (*Callithrix jacchus).*

**Methods:**

A total of fifty-four plasma samples were obtained from thirty-four marmosets that were inoculated with clinically-isolated DENV strains or DENV candidate vaccines, were used in this study. Plasma samples were obtained from marmosets after primary inoculation with DENV-2 infection, secondary inoculation with homologous or heterologous genotypes, and tertiary inoculation with heterologous DENV. Neutralizing antibody titers against DENV-2 (Cosmopolitan, Asian I, and Asian/American genotypes) and DENV-1 were determined using a conventional plaque reduction neutralization assay.

**Results:**

In marmosets that were inoculated with the Cosmopolitan genotype in primary infection, neutralizing antibody neutralized 3 genotypes, and the titers to Asian I genotype were significantly higher than those to homologous Cosmopolitan genotype. After secondary DENV-2 infection with heterologous genotype (Asian I in primary and Asian/American in secondary), neutralizing antibody titers to Asian/American genotype was significantly higher than those against Cosmopolitan and Asian I genotypes. Following tertiary infection with DENV-1 following DENV-2 Asian I and Cosmopolitan genotypes, neutralizing antibody titers to Asian/American were also significantly higher than those against Cosmopolitan and Asian I genotypes.

**Conclusion:**

The present study demonstrated that different levels of neutralizing antibodies were induced against variable DENV-2 genotypes after primary, secondary and tertiary infections, and that neutralizing antibody titers to some heterologous genotypes were higher than those to homologous genotypes within a serotype. The results indicate that heterogeneity and homogeneity of infecting genotypes influence the levels and cross-reactivity of neutralizing antibodies induced in following infections. The results also suggest that certain genotypes may possess advantage in terms of breakthrough infections against vaccination.

**Electronic supplementary material:**

The online version of this article (10.1186/s12985-018-0967-x) contains supplementary material, which is available to authorized users.

## Background

Dengue (DEN) is a major public health threat in tropical and subtropical regions, including Southeast Asia, the South Pacific, the Eastern Mediterranean, and the Americas [1, 2]. It is estimated that 50 to 100 million cases of dengue virus (DENV) infection occur annually worldwide. DENV is transmitted mainly by the *Aedes aegypti* and *Aedes albopictus* mosquitoes. The symptoms of DENV infection may range from undifferentiated viral fever, dengue without warning signs, and dengue with warning signs to life-threatening severe dengue [3]. There are four antigenically distinct serotypes of DENV, referred to as DENV1–4. There remains an urgent need for a high-efficacy dengue vaccine that offers long-term protection against all four DENV serotypes. All four DENV serotypes have similar clinical presentations and co-circulates in endemic areas. Among four serotypes, DENV-2 is the most prevalent serotype in the current worldwide dengue epidemics [4]. There are six genotypes of DENV-2, based on E-protein gene analysis: American, Asian/American, Asian I, Asian II, Cosmopolitan and Sylvatic [5, 6]. It is hypothesized that each DENV-2 genotype differs in terms of virulence and incidence [7].

Neutralizing antibodies play a central role in protection against DENV infection and disease pathogenesis [8]. Infection with one serotype of DENV confers life-long protection against the same serotype, but protection against infection with other serotypes is short-lived [9, 10]. DENV structural proteins, such as the E protein and the precursor membrane (pre-M), are the principal targets of antibody responses [11]. Because DENV serotypes are antigenically related, a majority of the antibody responses against the E-protein can cross-react with more than one DENV serotype [11, 12]. While serotype-specific neutralizing antibodies are induced after infection, these antibodies are highly genotype-specific, and strain variation within each serotype affects the ability of neutralizing antibodies to offer protection [6]. DENV- immunized mice have been shown to possess variable levels of neutralizing antibodies for different strains [13]. Currently, in DENV vaccine formulation, a single genotype is used for each serotype. Although genetic similarities among genotypes within a serotype are higher as compared to those among serotypes, the issue of differences in antigenicity among genotypes is likely to be critical in maximizing successful dengue vaccine development.

The objective of this research was to define genotype-specific and cross-reactive neutralizing antibody responses within a serotype. The common marmoset (*Callithrix jacchus*) model has been proved useful in primary and secondary DENV infection studies [14, 15]. The model demonstrates antibody response patterns consistent with those of human DENV infection and in candidate vaccines recipients [14–16]. In the present study, using this non-human primate model, we characterized in detail the neutralizing antibody responses to three DENV-2 genotypes in marmosets after primary, secondary and tertiary infections.

## Methods

### Viruses

Three strains comprised of DENV serotype-2 (DENV-2) genotypes were used in the study. DENV-2 DHF0663 strain (GenBank accession no. AB189122) was isolated from a DHF patient from Indonesia, and belonged to the Cosmopolitan genotype. DENV-2 00–43 strain (GenBank accession no. AB111452) was isolated from an imported dengue fever case from Indonesia, and belonged to the Asian I genotype. DENV-2 08–77 strain (GenBank accession no. AB545874) was isolated from an imported dengue fever case from the Maldives, and belonged to the Asian/American genotype (Additional file 1). These virus strains were first isolated using C6/36 cells, and passaged in baby hamster kidney (BHK) cells. Virus stocks were used within four cell culture passages. Culture supernatants collected from infected BHK cells were centrifuged at 3000 rpm for 5 min to remove cell debris. Virus stocks were stored at − 80 °C before use.

DENV-2 16,881 PDK-53 was a monovalent live attenuated DENV-2 vaccine candidate that were attenuated by serial passages of PDK cells (GenBank accession no. M84728). DENV-1 16,007 PDK-13 was a monovalent live attenuated DENV-1 vaccine candidate that were attenuated by serial passage of PDK cells (GenBank accession no. AF180818). All these vaccine strains were prepared at the Institute of Molecular Biosciences, Mahidol University, Nakhon Pathom, Thailand, shipped in liquid form on dry ice to National Institute of Infectious Diseases, Tokyo, Japan, and stored at − 80 °C before use. The DENV-1 and DENV-2 candidate vaccine and parent strains were kindly provided by Dr. Sutee Yoksan of Institute of Molecular Biosciences, Mahidol University, Thailand.

### Samples collected from marmosets

Common marmosets (*Callithrix jacchus)* were obtained from CLEA Japan, Inc. (Tokyo, Japan) and maintained in specific pathogen-free conditions at the National Institute of Infectious Diseases (Tokyo, Japan). A total of 54 blood samples were collected from 34 marmosets (21 females and 13 males) in the study. All marmosets used in this study were adult marmosets, with an age range of 4–8 years and weight range of 230–451 g. In groups 1–10, 38 plasma samples were collected from 26 marmosets with primary (*N* = 23), secondary (*N* = 12), and tertiary (*N* = 3) dengue virus infections (Table 1). In these group, each of the marmosets were inoculated subcutaneously on the back with 10^6^ plaque forming unit (PFU)/dose of DENV. The intervals between DENV inoculations were between 6 and 12 months. In groups 11–15, sixteen blood samples were collected from eight marmosets that were inoculated subcutaneously on the back with a monovalent live-attenuated dengue vaccine candidates: DENV-1 16,007 PDK-13 strain and DENV-2 16,881 PDK-53 strain (Table 1). In groups 11–15, marmosets received 10^4^ PFU/dose of DENV-1 and DENV-2 vaccine strains. About 6–9 months after the first administration, the marmosets were inoculated subcutaneously on the back with 10^5^ PFU/dose of DENV-1 or DENV-2. A total of 1 mL of whole blood was collected in EDTA tubes from each marmoset on day 0 (before the virus inoculation), and on days 4, 7, and 14 post-inoculation (p.i). Next, the blood samples were centrifuged at 2000 rpm for 10 min at 4 °C. The plasma samples were stored at − 80 °C before use. After primary inoculation, all marmosets were confirmed positive for DENV-specific IgM antibodies (Additional file 2).

**Table 1:** List of the marmosets that were used in this study inoculated with clinically isolated dengue virus

Type of infection	Primary infection	Secondary infection	Tertiary infection	Intervals between DENV infection	Marmosets ID
	Serotype (Genotype)	Serotype (Genotype)	Serotype (Genotype)		
Primary infection					
Group 1	D1 (GI)				M1, M2
Group 2	D1 (GII)				M3–1, M4–1, M5–1, M6,
Group 3	D2 (Cosmopolitan)				M7–1, M8–1, M9–1, M10, M11, M12, M13, M14
Group 4	D2 (Asian I)				M15–1, M16–1, M17–1, M18, M19, M20
Group 5	D2 (Asian/American)				M21, M22, M23
Secondary infection					
Homologous genotype					
Group 6	D2 (Cosmopolitan)	D2 (Cosmopolitan)		6 months	M24, M25
Heterologous genotype					
Group 7	D2 (Asian I)	D2 (Cosmopolitan)		6 months	M15–2, M16–2, M17–2, M26
Homologous serotype					
Group 8	D1 (GII)	D1 (GI)		7 months	M3–2, M4–2, M5–2
Heterologous serotype					
Group 9	D2 (Cosmopolitan)	D1 (GI)		12 months	M7–2, M8–2, M9–2
Tertiary infection					
Group 10	D2 (Asian I)	D2 (Cosmopolitan)	D1 (GI)	12 months	M15–3, M16–3, M17–3
Primary infection with monovalent live-attenuated DEN vaccine candidate					
Group 11	D1 16007 PDK-13 (GII)				V1–1, V2–1, V3–1, V4–1
Group 12	D2 16881 PDK-53 (Asian I)				V5–1, V6–1
Secondary infection					
Group 13	D1 16007 PDK-13 (GII)	D1 (GI)		7 months	V1–2, V2–2, V3–2, V4–2
Group 14	D2 16881 PDK-53 (Asian I)	D2 (Cosmopolitan)		6 months	V5–2, V6–2, V7–1, V8
Tertiary infection					
Group 15	D2 16881 PDK-53 (Asian I)	D2 (Cosmopolitan)	D1 (GI)	12 months	V5–3, V7–2

### Determination of neutralizing titer of the antibodies using a plaque reduction neutralization test assay

The neutralizing titers of the antibodies against the DENV-2 genotypes Cosmopolitan, Asian I, and Asian/American were determined using a plaque reduction neutralization test assay (PRNT). BHK cells were seeded in 12-wells plate in Minimum Essential Medium (MEM) (Sigma Aldrich, USA) supplemented with 10% of heat-inactivated fetal bovine serum (Hi-FBS) (Thermo Fisher Scientific, USA)) and were incubated at 37 °C overnight until the cells monolayer reached approximately 70% confluency. Plasma samples were heat-inactivated at 56 °C for 30 min before use. The heat-inactivated plasma samples were serially diluted two-fold starting from 1:10 to 1:20,480 in MEM supplemented with 10% Hi-FBS. The virus-antibody complexes were prepared by mixing 25 μL of DENV at titers of 5000 PFU/ml with 25 μL serially diluted plasma samples to make a final dilution series ranging from 1:20 to 1:40, 960. The control samples were prepared by mixing 25 μL DENV at titers of 5000 PFU/ml with 25 μL MEM supplemented with 10% Hi-FBS. Next, the virus-antibody complexes were incubated at 37 °C for 60 min. A total of 50 μL of the mixture was then inoculated onto BHK cell monolayers in 12-wells plates. After incubation for 60 min, 1 mL of maintenance medium containing MEM and 1% of methyl cellulose supplemented with 2% of Hi-FBS was added. The plates were incubated at 37 °C in 5% CO_2_ until visible plaques were observed (5–7 days of incubation). Cells were fixed with 10% formaldehyde and stained with methylene blue, washed with water, and then the number of plaques was counted. All tests were conducted in duplicate. The neutralizing antibody titer was expressed as the maximum dilution of plasma sample that yielded a ≥ 50% plaque reduction in the virus inoculum compared with control virus samples. Results are shown as 50% PRNT_50_ values, expressed as the reciprocal of the highest plasma dilution (end-point titer) that results in ≤50% of the input plaque count.

### Data analysis

Data were analyzed using the statistical analysis toolpack in Microsoft Excel 2016 (Microsoft Corporation, USA) and GraphPad Prism 7 (GraphPad Software Inc., USA). Student’s test was performed to compare the mean titers of neutralizing antibodies. *p* values less than 0.05 were considered statistically significant.

### Ethics statement

The animal studies were conducted in accordance with the guidelines of the Institutional Animal Care and Use Committee of the National Institute of Infectious Diseases (NIID), Tokyo, Japan. The study was approved by the Institutional Animal Care and Use Committee of NIID (approval no. 613006 and 516,010). All animal and infection experiments were performed according to the NIID Institutional Guidelines, in additions to the guidelines of the Science Council, and local rules and regulations.

## Results

### Levels of neutralizing antibodies to different DENV-2 genotypes following primary infection

**Fig. 1:**
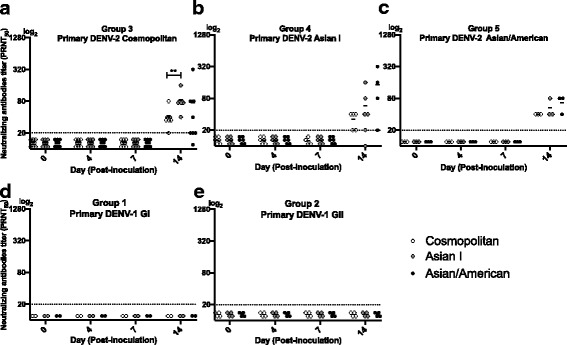
Neutralizing antibodies titers to DENV-2 genotypes (Cosmopolitan, Asian I and Asian/American) during primary DENV inoculation. Neutralizing antibody titers in marmosets inoculated with: **a** DENV-2 genotype Cosmopolitan, **b** DENV-2 genotype Asian I, **c** DENV-2 genotype Asian/American, **d** DENV-1 genotype I, **e** DENV-1 genotype II. Double asterisks (**) indicate that there were statistically significant differences (*p* < 0.05) in the titers of neutralizing antibodies between genotypes. Student’s test analysis was used to determine the differences in mean titers of neutralizing antibody between CM vs AI, CM vs AA, and AI vs AA. CM indicates Cosmopolitan genotype. A1 indicates Asian I genotype. AA indicates Asian/American genotype

Marmosets were inoculated with one of 3 genotypes of DENV-2, and neutralizing antibody titers were examined to homologous and heterologous genotypes. Neutralizing antibodies were not detected to any of the 3 genotypes (Cosmopolitan, Asian I, and Asian/America genotypes) from days 0 to 7 post-inoculation (p.i) (Fig. 1a-c). However, the marmosets inoculated with Cosmopolitan, Asian I, and Asian/American genotypes (*N* = 23) presented neutralizing antibodies to the Cosmopolitan, Asian I, and Asian/American genotypes by day 14 p.i (Fig. 1a-c). In eight marmosets that were inoculated with the Cosmopolitan genotype (Group 3), titers of neutralizing antibody to the Asian I genotype were significantly higher as compared to those for the Cosmopolitan genotype (*p* = 0.007) on the day 14 p.i (Fig. 1a). In marmosets inoculated with the Asian I (group 4) or Asian/American (group 5) genotypes, neutralizing antibody was also detected to the 3 genotypes, but the titers were found to be at similar levels among these genotypes (Fig. 1b-c). In contrast, in the primary DENV-1 infection (group 1 and group 2), neutralizing antibodies to Cosmopolitan, Asian I, and Asian/American genotypes of DENV-2 were absent (< 20) on days 0, as well as on days 4, 7, and 14 p.i. (Fig. 1d-e). The results confirm that neutralizing antibodies induced by primary infection with DENV-2 do not neutralize heterologous infection with DENV-1.

### Neutralizing antibody responses after secondary DENV-2 infection in marmosets which were previously infected with homologous or heterologous DENV-2 genotype

**Fig. 2:**
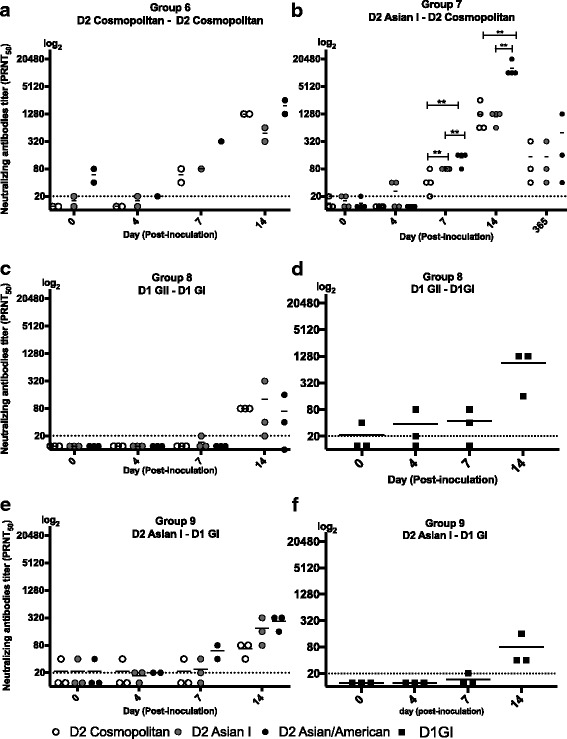
Neutralizing antibodies titers to DENV-2 genotypes (Cosmopolitan, Asian I and Asian/American) after secondary DENV inoculation. Neutralizing antibody titers in marmosets inoculated with: **a** Homologous DENV-2 genotype(Cosmopolitan), **b** Heterologous DENV-2 genotypes (Asian1 in primary, Cosmopolitan in secondary), **c** two DENV-1 genotypes (Genotype 1 in primary, Genotype II in secondary), **d** Neutralizing antibody titers to DENV-1 genotype I during two DENV-1 genotypes (Genotype 1 in primary, Genotype II in secondary), **e** Heterologous serotypes (DENV-2 Asian1 genotype in primary, DENV-1 Genotype 1 in secondary), **f** Neutralizing antibody titers to DENV-1 genotype I after inoculation with heterologous serotypes (DENV-2 Asian1 genotype in primary, DENV-1 Genotype 1 in secondary). Double asterisks (**) indicates that there were statistically significant differences (*p* < 0.05) in the titers of neutralizing antibodies between genotypes. Student’s test analysis was used to determine the differences in mean titers of neutralizing antibody between CM vs AI, CM vs AA, and AI vs AA. Neutralizing antibody data at day 365 post-inoculation of (**b**) are the same data as day0 of Fig. 4b. CM indicates Cosmopolitan genotype, A1 indicates Asian I genotype and AA indicates Asian/American genotype

Marmosets which had been previously infected with DENV-2 were infected with homologous and heterologous genotypes (Fig. 2a and b). Prior to secondary infection with homologous genotype (group 6), neutralizing antibody titers to Asian/American genotypes were detected at day 0, indicating that neutralizing antibody induced during primary infection cross-neutralize different genotypes within the same infecting serotype. Additionally, neutralization antibody levels to Asian/American genotype was higher as compared to the infecting genotype (Cosmopolitan genotype), after primary infection. The results suggest that while antibodies neutralize different genotypes within a serotype, the levels varies among genotypes. The result indicate that after secondary infection with homologous genotype (group 6), neutralizing antibody titers rapidly increased from day 7, but titers did not differ significantly among 3 genotypes (Cosmopolitan, Asian I and Asian/American) at day 14 p.i. (Fig. 2a).

After secondary DENV-2 infection with heterologous genotype (group 7), levels of neutralizing antibody rapidly increased from day 7. The titers of neutralizing antibody to Asian/American genotype were significantly higher as compared to those against the Cosmopolitan (*p* = 0.029) and Asian I (*p* = 0.020) genotypes at day 7 p.i. Titers of neutralizing antibody to the Asian I genotype were also significantly higher than those against Cosmopolitan (*p* = 0.034) at day 7. Similarly, at day 14 p.i, the neutralizing antibody titers against the Asian/American genotype were significantly higher as compared to titers against the Cosmopolitan (*p* = 0.010) and Asian I (*p* = 0.009) genotypes. It is of interest that the antibodies possessed higher neutralization activities towards the Asian/American genotype, which were not uses in primary and secondary infection, than towards the Cosmopolitan and Asian I genotypes. Neutralizing antibody titers against the Asian I and Asian/American genotypes after secondary infection with heterologous genotype (Asian I-Cosmopolitan) (Fig. 2b) were significantly higher as compared to those against homologous genotype (Cosmopolitan-Cosmopolitan) secondary infection (Fig. 2a); (*p* = 0.030) and (*p* = 0.023), respectively. These results suggest that higher neutralizing activity was induced after secondary infection with heterologous genotype. At day 365 p.i (day 0 prior to third challenge; Figs. 2b and 4c), the levels of neutralizing antibodies to Cosmopolitan, Asian I, and Asian/American were still detected but there were no significant differences between neutralizing activity amongst the genotypes.

### Neutralizing antibody responses to DENV-2 after secondary infection with DENV-1 in marmosets which were previously infected with DENV-1

Marmosets in group 8 which had been previously infected with DENV-1 genotype II were infected with heterologous genotype I of DENV-1, and neutralizing antibody titers to 3 genotypes of DENV-2 were examined (Fig. 2c and d).

Neutralizing antibodies titers against 3 DENV-2 genotypes (Cosmopolitan, Asian I, and Asian/American genotypes) were below the detection levels at days 0, 4, and 7 p.i (Fig. 2c). However, the marmosets demonstrated neutralizing activities to three DENV-2 genotypes at day 14 p.i, and the titers were at similar levels. Neutralizing antibody to DENV-1 were detected in all the marmosets on day 14 (Fig. 2d). The results indicate that two inoculations with DENV-1 induced antibodies which neutralized 3 genotypes (Cosmopolitan, Asian I and Asian/American) of heterologous serotype (DENV-2) (Fig. 2c), as well as homologous serotype (DENV-1) (Fig. 2d).

### Neutralizing antibody responses after secondary DENV-1 infection in marmosets which were previously infected with DENV-2

Marmosets in group 9 which had been previously infected with DENV-2 Asian I genotype were infected with DENV-1, and neutralizing antibody titers to 3 genotypes of DENV-2 were examined (Fig. 2e and f).

One marmoset demonstrated neutralizing antibodies to Cosmopolitan, Asian I, and Asian/American genotypes prior to dengue infection at day 0 (Fig. 2e). Neutralizing antibodies titers to Cosmopolitan, Asian I, and Asian/American genotypes increased in marmosets from days 7 p.i, and there were no significant differences in neutralizing antibody titers for these different genotypes (Fig. 2e). Neutralizing antibodies to the DENV-1 serotype were detected by day 7 p.i (Fig. 2f). Levels of neutralizing antibodies to the Asian/American genotype after two inoculations with homologous (DENV-2) and heterologous (DENV-1) serotypes were significantly higher than those after the two inoculations with DENV-1 (*p* = 0.020) (Fig. 2c).

### The neutralizing antibody response to DENV-2 genotypes in marmosets inoculated with DENV-1 and DENV-2 candidate vaccine

**Fig. 3:**
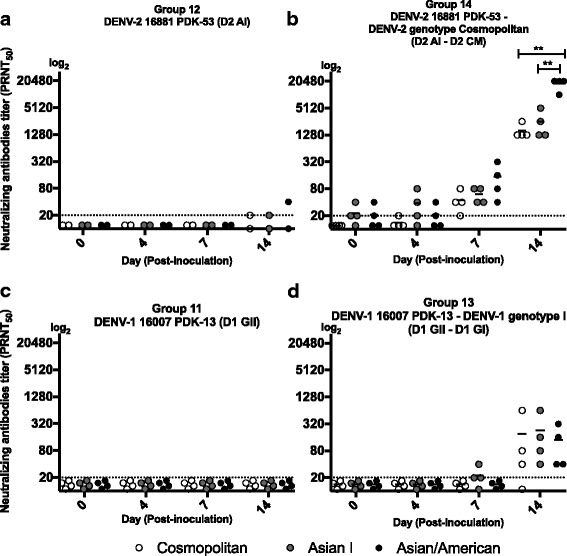
Neutralizing antibodies titers to DENV-2 genotypes (Cosmopolitan, Asian I and Asian/American) in marmosets inoculated with candidate dengue vaccines and challenged with isolated DENV. Neutralizing antibody titers in marmosets inoculated with: **a** Candidate vaccine DENV-2 16,881 PDK-53, **b** Candidate vaccine DENV-2 16,881 PDK-53 in primary and with DENV-2 genotype Cosmopolitan in secondary, **c** Vaccine candidate DENV-1 16,007 PDK-13, **d** Vaccine candidate DENV-1 16,007 PDK-13 in primary and DENV-1 genotype I in secondary. Double asterisks (**) indicate that there were statistically significant differences (*p* < 0.05) in the titers of neutralizing antibodies between genotypes. Student’s test analysis was used to determine the differences in mean titers of neutralizing antibody between CM vs AI, CM vs AA, and AI vs AA. CM indicates Cosmopolitan genotype. A1 indicates Asian I genotype. AA indicates Asian/American genotype

In this series of experiments, we used live-attenuated vaccine candidate to determine whether similar pattern of genotype cross-reactivity is induced by vaccine candidates. Marmosets were immunized with DENV-2 16,881 PDK-53 (Asian I genotype) and challenged with DENV-2 Cosmopolitan genotype. Neutralizing antibody titers to Cosmopolitan, Asian I and Asian/American genotypes were examined. One of two marmoset that was inoculated with the DENV-2 16,881 PDK-53 candidate vaccine (Asian I genotype), however, presented neutralizing antibodies to DENV-2 Cosmopolitan, Asian I, and Asian/American at the day 14 p.i (Fig. 3a). After challenge (secondary infection) with DENV-2 Cosmopolitan genotype, neutralizing antibodies were detected to 3 genotypes in all the 4 marmosets on day 7 and day 14 p.i (Fig. 3b). Neutralizing antibody titers against the Asian/American genotype were also significantly higher compared to those against the Cosmopolitan and Asian I genotypes at the day 14 p.i (*p* = 0.005 and *p* = 0.003, respectively).

Other group of marmosets were immunized with DENV-1 16,007 PDK-13 (genotype II) and challenged with DENV-1 genotype I. Neutralizing antibody activity was not detected on days 0–14 after inoculation with DENV-1 16,007 PDK-13 (Fig. 3c). After challenge with DENV-1 genotype I (secondary infection), serotype cross-reactive antibodies to three genotypes of DENV-2 (Cosmopolitan, Asian I, and Asian/American genotype) were detected at day 14 p.i (Fig. 3d). The results are consistent with shown in Fig. 2c, and indicate that two inoculations with DENV-1 induced antibodies which neutralized 3 genotypes of heterologous serotype (DENV-2) as well as homologous serotype.

### Neutralizing antibody responses after tertiary DENV infection

**Fig. 4:**
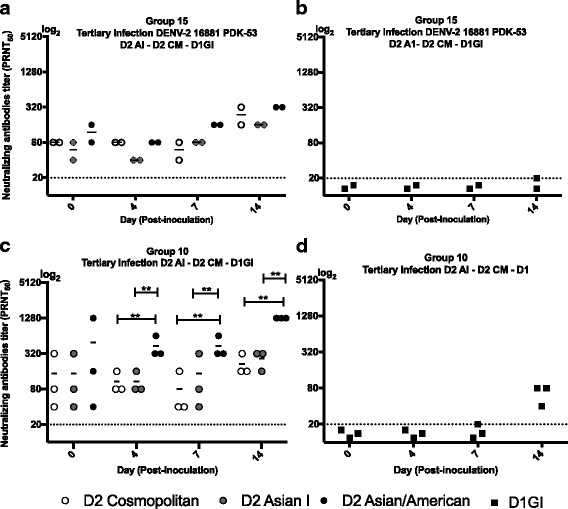
Neutralizing antibodies titers to DENV-2 genotypes (Cosmopolitan, Asian I and Asian/American) after tertiary inoculation. **a** Marmosets which had been first inoculated with DENV-2 16,881 PDK-53 (Asian I genotype) and then challenged with DENV-2 genotype Cosmopolitan were infected with DENV-1 in tertiary inoculation. **b** Neutralizing antibody titers to DENV-1, genotype 1 in the same marmosets in (**a**). **c** Marmosets that were first inoculated with the DENV-2 Asian I genotype and then challenged with the DENV-2 genotype Cosmopolitan were infected with DENV-1 genotype I in tertiary inoculation. **d** Neutralizing antibody titers to DENV-1, genotype 1 in the same marmosets in (**c**). Double asterisks (**) indicate that there were statistically significant differences (*p* < 0.05) in the titers of neutralizing antibodies between genotypes. Student’s test analysis was used to determine the differences in mean titers of neutralizing antibody between CM vs AI, CM vs AA, and AI vs AA. CM indicates Cosmopolitan genotype. A1 indicates Asian I genotype. AA indicates Asian/American genotype

The neutralizing antibody responses to 3 genotypes of DENV-1 were also examined after tertiary DENV inoculation (group 10 and group 15). Marmosets which had been first inoculated with DENV-2 16,881 PDK-53 (Asian I genotype) and then challenged with DENV-2 genotype Cosmopolitan were infected with DENV-1 in tertiary inoculation (Fig. 4a). Neutralizing antibody to Cosmopolitan, Asian I and Asian/American genotypes were detected from day 0 p.i. The levels increased by day 14, but the titers were at similar levels amongst 3 genotypes of DENV-2 (40–320, *p* = 0.250). Neutralizing activity to DENV-1 genotype 1 was not detected except for one marmosets on day 14 (Fig. 4b).

Three marmosets that were first inoculated with the DENV-2 Asian I genotype and then challenged with the DENV-2 genotype Cosmopolitan were infected with DENV-1 genotype I in tertiary inoculation (Fig. 4c). Neutralizing antibody to Cosmopolitan, Asian I and Asian/American genotypes were detected prior to tertiary virus inoculation. The levels of neutralizing antibody to the Asian/American genotype increased and the titers were higher compared to those against the Cosmopolitan and Asian I genotypes at days 4, 7, and 14 p.i (p_day4_ = 0.029, p_day7_ = 0.017, and p_day14_ = 0.001 for the Cosmopolitan genotype, and p_day4_ = 0.029, p_day7_ = 0.034, and p_day14_ = 0.001 for the Asian I genotype). These marmosets (*n* = 3) demonstrated DENV-1 neutralizing antibodies at PRNT_50_ titers between 40 and 80 at day 14 p.i (Fig. 4d).

## Discussion

It has been reported that after primary infection, serotype cross-reactive anti-E antibodies were induced, and neutralizing antibody levels were the highest for the infecting homologous serotype [17–20]. In the present study, we found that neutralizing antibodies induced during primary infection with a single genotype neutralized multiple genotypes within DENV-2. The levels of neutralizing antibody to the 3 genotypes of DENV-2 (Cosmopolitan, Asian I, and Asian/American genotypes) (group 4, Fig. 1b) were decreased 6-months after the primary infection (Fig. 2b, day 0) and was consistent with other studies using samples from non-human primates and human [21].

We found that marmosets that were inoculated with heterologous genotype/serotype infection had variable levels of neutralizing antibody in response to 3 different genotype of DENV-2 (Cosmopolitan, Asian I, and Asian/American genotypes) as compared to those marmosets with homologous genotype infection. In marmosets that were inoculated with secondary homologous genotype infection, neutralizing antibodies neutralized Cosmopolitan, Asian I and Asian/American genotypes at similar levels. Interestingly, in marmosets with the secondary heterologous genotype infection, levels of neutralizing antibodies to heterologous Asian/American genotype were significantly higher as compared to those against the Cosmopolitan and Asian I genotypes. Similar results were also observed during the tertiary heterologous serotype infection indicating that cross-neutralizing antibody responses consistently recognized similar antigens even though the marmosets were not inoculated with Asian/American genotypes [21]. In marmosets inoculated with DENV-2 vaccine candidate (Asian I genotype) and infected with Cosmopolitan genotype, antibody neutralized three genotypes of DENV-2 (Cosmopolitan, Asian I, and Asian/American), but neutralizing titers to Asian/American genotype was significantly higher than those to the Cosmopolitan and Asian I genotypes (Fig. 3b). These results are consistent with those obtained using isolated strains shown in Fig. 2b. In our study, the differences in the levels of neutralizing antibodies against 3 genotypes were most prominent when antibody titers were at high levels (PRNT_50_ = 1:640–20,480). Of note, although the differences were not significant in the primary infection with Asian 1 genotype, inoculation with heterologous genotype (Cosmopolitan) in the secondary infection induced high levels of antibodies that significantly better neutralized the Asian/American genotype than Asian I and Cosmopolitan genotypes. This pattern was not observed after two infections with homologous genotype (Cosmopolitan genotype) (Fig. 2a) and after two infections with heterologous serotypes (DENV-2 Asian 1 and DENV-1 genotype 1) (Fig. 2e). These results suggest that heterogeneity in antigenic molecules between genotypes may lead to the induction of higher levels of antibody to the epitope of the third genotype (Asian/American in the experiment), or synergize in neutralization of the third virus. However, elevated levels of neutralizing antibodies to the Asian/American genotype were not observed one year after secondary infection, when the levels of neutralizing antibodies decreased. These data suggest the presence of a subset of antibodies that better neutralize the Asian/American genotype, and that the subset and neutralizing effects were only observed when the overall neutralizing antibody activities were at high levels (Figs. 2b and 4c).

The marmosets presented neutralizing antibodies to DENV-2 genotypes after two infections with DENV-1 (DENV-1 GII in primary and DENV-1 GI in secondary infections) (Fig. 2c). These data suggest that serotype cross-reactive neutralizing antibodies are induced to some degrees, and that a robust secondary homologous immune response (mean secondary homologous DENV-1 PRNT_50_ = 1:907; primary DENV-1 PRNT_50_ = 1:60) may make serotype cross-reactive neutralizing antibody response more apparent. While infection with a single serotype typically leads to high levels of serotype-specific antibodies, it is likely that by two infections with homologous serotype, serotype cross-reactive antibodies may be boosted over threshold levels and are able to cross-neutralize other serotypes. In this regard, secondary homologous infection may partially contribute to serotype cross-protection in residents of DENV hyperendemic areas, due to a higher risk of repeated exposure to the same serotype. The next step would be to further expand our understanding of the spectrum of the neutralizing antibody patterns after heterologous and homologous infection, using different genotype and serotype combinations.

Cross-reactive neutralizing antibodies induced during sequential infection with different serotypes generally possess broad cross-reactivity and neutralizes multiple serotypes including non-infecting serotypes [19, 22, 23]. Previous studies from other groups on DEN cohort patients demonstrated that after heterologous secondary infection, neutralizing antibodies cross-react and neutralize multiple serotypes [24, 25] Notably, marmosets with heterologous serotype infection (primary DENV-2, secondary DENV-1) presented higher levels of neutralizing antibodies to previously infecting serotypes, compared to those against the secondary serotype. Studies examining maternal DENV antibodies demonstrated that the antibody decay could lead to partial protection and virus infection-enhancement [26, 27]. Similarly, although high levels of neutralizing antibodies to the previous infecting serotypes persisted for up to nine months after sequential infection, the antibody levels in marmosets decreased with time. Furthermore, the subset of antibodies that better neutralize the Asian/American genotype were diluted over time. Because this study was conducted based on one single technique and an animal model, further studies would be needed to address the correlation between levels of neutralizing antibody and protection against DENV infection. Previous studies have reported that during secondary homologous serotype infection, the marmosets were protected against re-infection with same serotype of DENV [14, 28]. In secondary heterologous serotype infection and tertiary heterologous infection, levels of viremia (4 × 10^2^ – 2 × 10^5^ pfu/ml) were still detected in the marmosets in groups 9, 10, and 15, at day 4–7 p.i when infected with different serotype of DENV (unpublished results). Thus, further studies using a larger set of data is expected to address the levels of neutralizing antibodies and protection against infection with heterologous serotypes.

Overall, this study highlighted that antibodies neutralize different genotypes within infecting serotypes, and that the levels were significantly different among different genotypes. While clinical trials of candidate vaccines demonstrated that vaccines are immunogenic in both humans and animal models, there are limited data available on the variability of neutralizing activities against different genotypes within serotype. Our study demonstrate that neutralizing antibodies induced by the Asian I and Cosmopolitan genotypes possessed higher neutralizing activities against the Asian/American genotype than against the homologous genotypes: Cosmopolitan and Asian I. The variability in the neutralizing antibodies titers between genotypes suggest that sequence heterogeneity between genotypes could result in complex genotype cross-reactive immune responses; leading to distinct neutralizing antibody patterns among the different genotypes [23, 29–32]. Similarly, marmosets inoculated with a candidate vaccine demonstrated variability in their neutralizing antibody responses comparable to those induced by the parental strain; suggesting that while attenuation leads to decreased pathogenicity, immunogenicity patterns between genotypes are retained. Thus, determination of the spectrum of neutralizing activity in marmosets in relation to the ability to offer protection to multiple serotypes and genotypes offers deep insights into dengue vaccine efficacy issues and disease control.

## Conclusion

The present study demonstrated that different levels of neutralizing antibodies were induced against various DENV-2 genotypes after primary and secondary infections and, that neutralizing antibody titers to certain heterologous genotype were higher than those to homologous genotypes within a serotype. The result indicates that heterogeneity and homogeneity of genotypes in primary and secondary infections influence the levels and cross-reactivity of neutralizing antibodies induced after secondary infections. The results also suggest that certain genotypes may possess advantage in terms of breakthrough infections against dengue vaccination.

## Additional files

Additional file 1: Phylogenetic tree of the dengue virus serotype 2 strains that were used in this study. The phylogenetic tree was constructed by using the nucleotide sequence of the complete envelope protein region. Closed arrows indicates virus strains that were used in this study, DENV-2 strain DHF0663 belongs to genotype Cosmopolitan, DENV-2 strain 00–43 belongs to genotype Asian I, and DENV-2 strain 08–77 belongs to genotype Asian/American. (PDF 48 kb)

Additional file 2: Levels of IgM antibody in plasma samples of the marmosets according to the group and type of infection. The levels of IgM antibody were determined using IgM ELISA. The positive detection of IgM is determined when the positive/negative ratio ≥ 2.0. Dash lines indicate the baseline of the positive detection of IgM antibody. (PDF 57 kb)

## Abbreviations

AA: Dengue virus 2 genotype Asian/American
AI: Dengue virus 2 genotype Asian I
CM: Dengue virus 2 genotype Cosmopolitan
DENV: Dengue virus
p.i: Post-inoculation
PRNT: Plaque reduction neutralization test
