# Supplementary figures and images for: Genotype-specific and cross-reactive neutralizing antibodies induced by dengue virus infection: detection of antibodies with different levels of neutralizing activities against homologous and heterologous genotypes of dengue virus type 2 in common marmosets (Callithrix jacchus)
Source: Virol J. 2018 Mar 27;15:51. doi: 10.1186/s12985-018-0967-x (PMC5870686; doi:10.1186/s12985-018-0967-x)

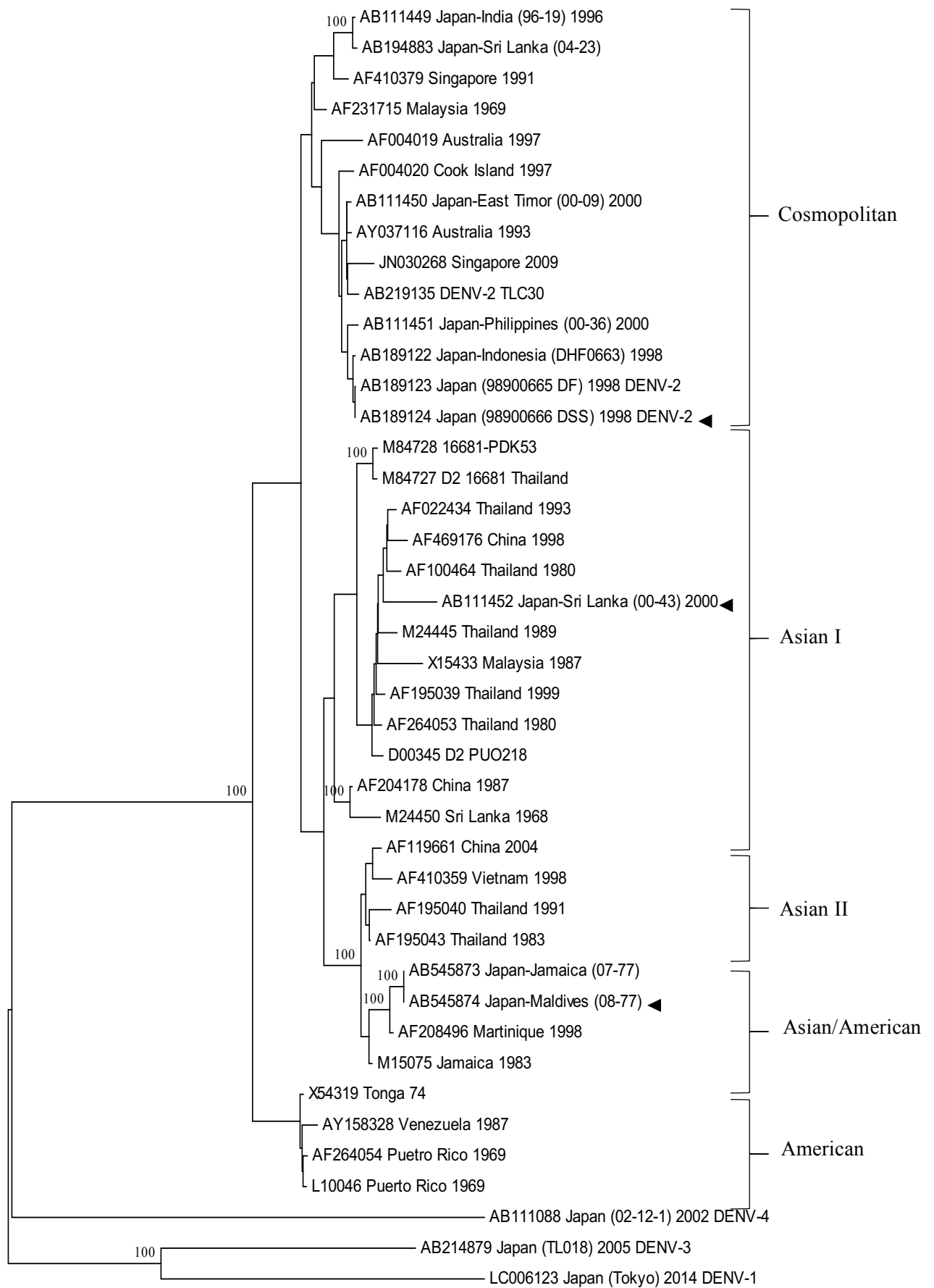

Supplement: Supplementary file 1 — Phylogenetic tree of the dengue virus serotype 2 strains that were used in this study. The phylogenetic tree was constructed by using the nucleotide sequence of the complete envelope protein region. Closed arrows indicates virus strains that were used in this study, DENV-2 strain DHF0663 belongs to genotype Cosmopolitan, DENV-2 strain 00–43 belongs to genotype Asian I, and DENV-2 strain 08–77 belongs to genotype Asian/American. (PDF 48 kb) [file 12985_2018_967_MOESM1_ESM.pdf]

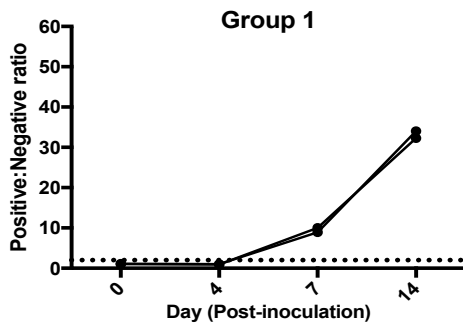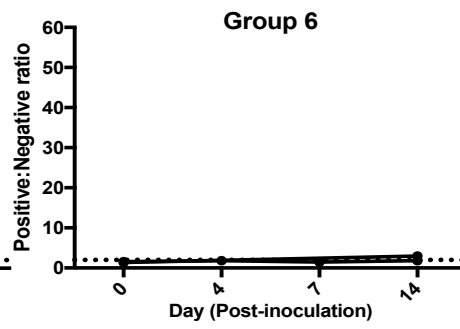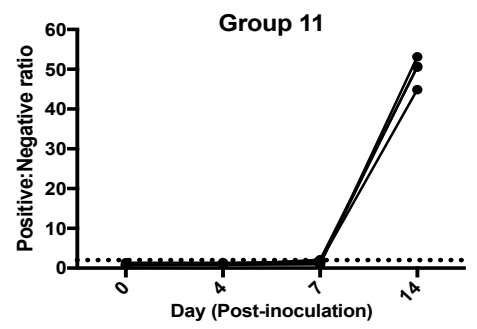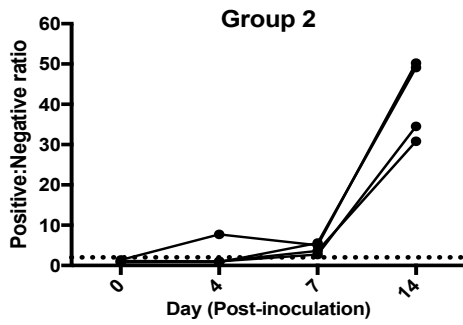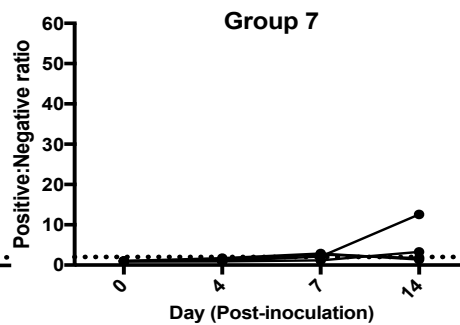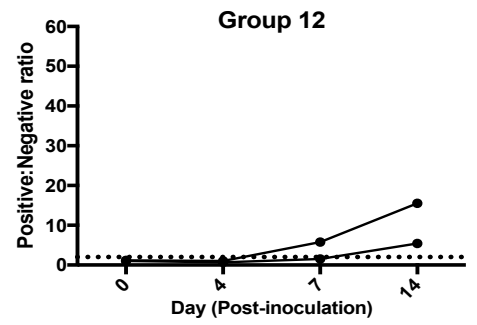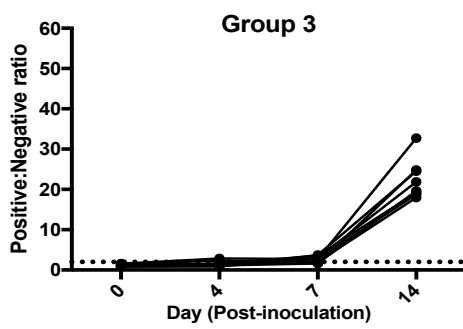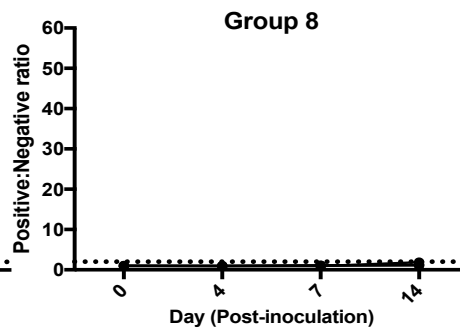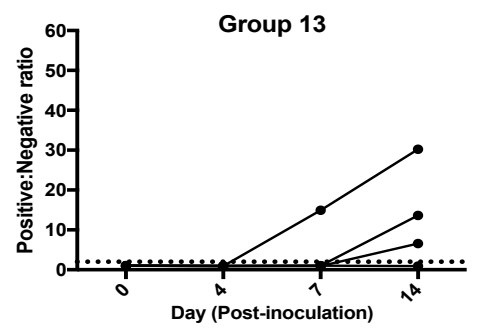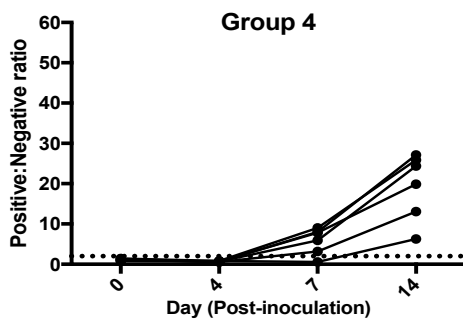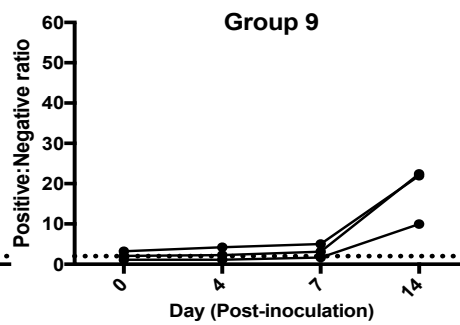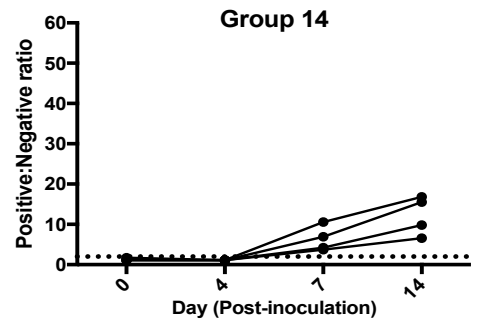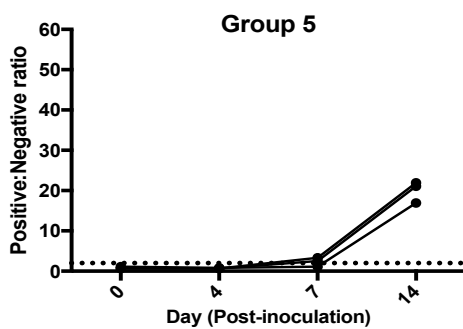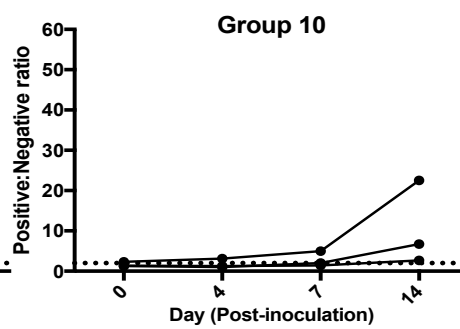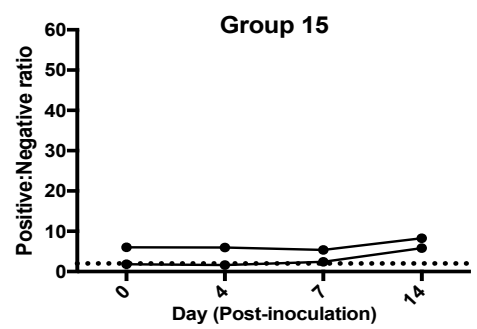

Supplement: Supplementary file 2 — Levels of IgM antibody in plasma samples of the marmosets according to the group and type of infection. The levels of IgM antibody were determined using IgM ELISA. The positive detection of IgM is determined when the positive/negative ratio ≥ 2.0. Dash lines indicate the baseline of the positive detection of IgM antibody. (PDF 57 kb) [file 12985_2018_967_MOESM2_ESM.pdf]
